# Supplementary material for: High Clinical Value of Liquid Biopsy to Detect Circulating Tumor Cells and Tumor Exosomes in Pancreatic Ductal Adenocarcinoma Patients Eligible for Up-Front Surgery
Source: Cancers (Basel). 2019 Oct 26;11(11):1656. doi: 10.3390/cancers11111656 (PMC6895804; doi:10.3390/cancers11111656)
Supplement: Supplementary file 1 [file cancers-11-01656-s001.pdf]

Supplementary Materials: High Clinical Value of Liquid Biopsy to Detect Circulating Tumor Cells and Tumor Exosomes in Pancreatic Ductal Adenocarcinoma Patients Eligible for Up-Front Surgery

Etienne Buscail, Catherine Alix-Panabières, Pascaline Quincy, Thomas Cauvin, Alexandre Chauvet, Olivier Degrandi, Charline Caumont, Séverine Verdon, Isabelle Lamrissi, Isabelle Moranvillier, Camille Buscail, Marion Marty, Christophe Laurent, Véronique Vendrely, François Moreau-Gaudry, Aurélie Bedel, Sandrine Dabernat and Laurence Chiche

Table S1. Clinical features of the cohort.

Variables	PDAC Group (*n* = 22) (%)	Control GROUP (*n* = 28) (%)
Age, year, (median; range)	68.8 (69.5; 57–81) *	58.3 (61;22–73) *
Male gender, *n* (%)	13 (59)	8 (28)
BMI (median; range)	24(25;18–35)	26.5(25; 18–45)
ASA score		
1–2	16 (72)	19 (67)
≥3	6 (28)	9 (33)
Smoking status	5 (22)	12 (42)
First symptoms		IPMN (*n* = 8)
Acute pancreatitis	0 (0)	2(25)
Jaundice	13 (59)	0(0)
New onset of diabetes	0 (0)	1(12.5)
Weight loss	5 (22)	0(0)
Diarrhea and/or steatorrhea	0 (0)	0(0)
Abdominal pain	3 (13)	3(37.5)
No symptoms	4 (18)	3(37.5)
Serum CA 19-9		
Normal	16 (72)	23 (83)
Elevated	6 (28)	5 (17)
EUS-FNA	Total *n* = 13	
Positive	*n* = 7 (54)	Positive *n* = 0 (0)
Negative	*n* = 6 (46)	Negative *n* = 3 (100)

* Statistically significant; IPMN are included in the control group. Abbreviations: med, median; PDAC, pancreatic ductal adenocarcinoma; IPMN, intraductal papillary and mucinous neoplasm; EUS-FNA, endoscopic ultrasound-guided fine needle aspiration; BMI, Body mass index; ASA, American society of anesthesiologists. Serum CA 19-9 was considered as elevated when greater than 37 UI/mL.

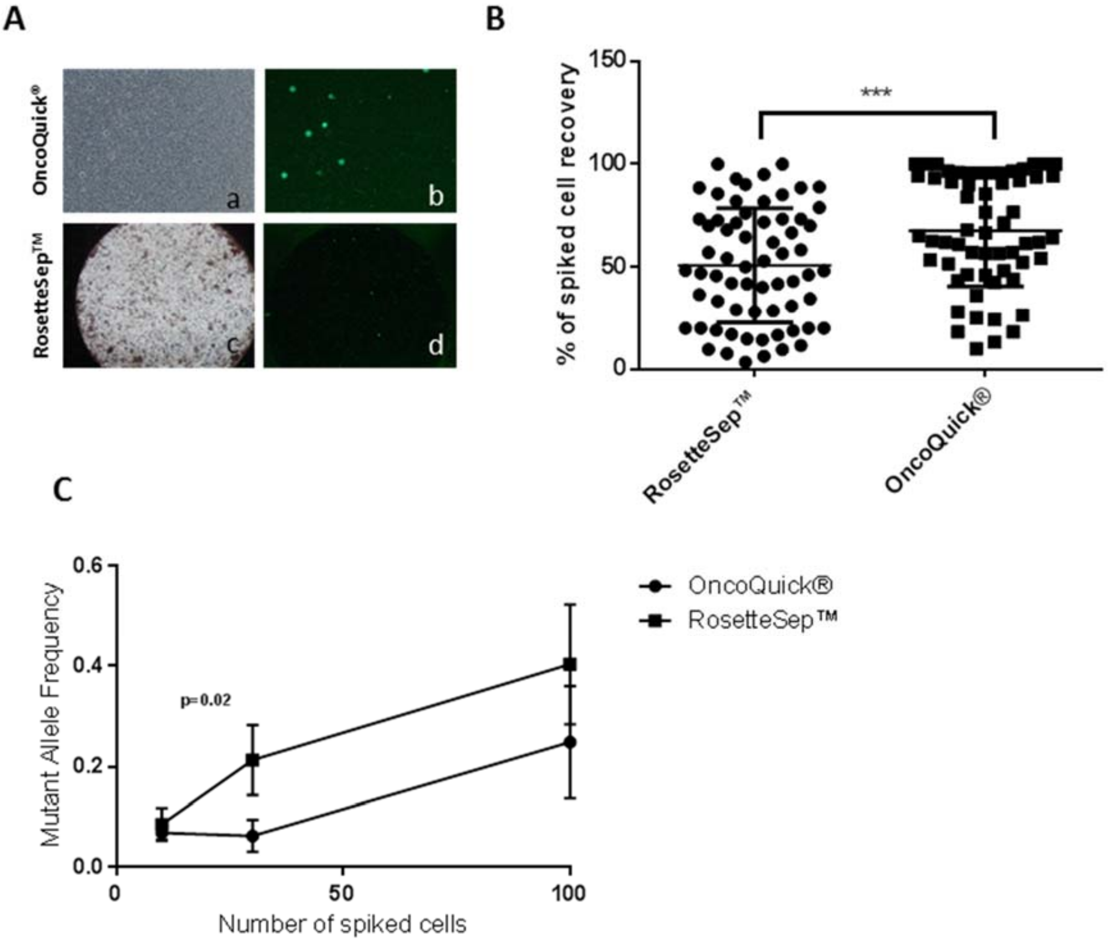

Figure S1. Tumor cell detection in cell spiking experiments. (**A**). 60-well plate pictures after fluorescent cell spiking and recovery images of tumor cells obtained after OncoQuick® enrichment (a, bright field; b, fluorescence) and RosetteSep™ enrichment (c, bright field; d, fluorescence) all images with original magnification ×100. (**B**). Recovery efficiency for two methods. ***: $p < 0.001$: Cell count and tumor cell recovery after spiking experiments. Statistically greater count in Oncoquick® compared with RosetteSep™ paired analysis performed by Wilcoxon test ($p < 0.001$). (**C**). Mutant allele frequency determined by ddPCR after enrichment according to spiked cells: Molecular detection of mutant *K-RAS* alleles by ddPCR after RosetteSep™ enrichment was 3- to 4-fold more sensitive than after OncoQuick®.

© 2019 by the authors. Licensee MDPI, Basel, Switzerland. This article is an open access article distributed under the terms and conditions of the Creative Commons Attribution (CC BY) license (http://creativecommons.org/licenses/by/4.0/).
